# A Datasheet for the INSIGHT Birmingham, Solihull, and Black Country Diabetic Retinopathy Screening Dataset

**DOI:** 10.1016/j.xops.2023.100293

**Published:** 2023-02-26

**Authors:** Aditya U. Kale, Andrew Mills, Emily Guggenheim, David Gee, Samuel Bodza, Aparna Anumakonda, Rima Doal, Rowena Williams, Suzy Gallier, Wen Hwa Lee, Paul Galsworthy, Manjit Benning, Hilary Fanning, Pearse A. Keane, Alastair K. Denniston, Susan P. Mollan

**Affiliations:** 1 Academic Unit of Ophthalmology, Institute of Inflammation & Ageing, College of Medical and Dental Sciences, University of Birmingham, Birmingham, UK; 2 Ophthalmology Department, University Hospitals Birmingham NHS Foundation Trust, Birmingham, UK; 3 Birmingham Heartlands Hospital, University Hospitals Birmingham NHS Foundation Trust, Birmingham, UK; 4 Health Informatics, University Hospitals Birmingham NHS Foundation Trust, Birmingham, UK; 5 INSIGHT Health Data Research Hub for Eye Health, United Kingdom; 6 Action Against Age-Related Macular Degeneration, London, UK; 7 Moorfields Research & Development, Moorfields Eye Hospital NHS Foundation Trust, London, UK; 8 Research and Development, University Hospitals Birmingham NHS Foundation Trust, Birmingham, UK; 9 NIHR Biomedical Research Centre at Moorfields Eye Hospital NHS Foundation Trust, UCL Institute of Ophthalmology, London, UK

**Keywords:** Diabetic retinopathy, Biomedical data, Dataset, Diabetes mellitus, Imaging

## Abstract

**Purpose:**

Diabetic retinopathy (DR) is the most common microvascular complication associated with diabetes mellitus (DM), affecting approximately 40% of this patient population. Early detection of DR is vital to ensure monitoring of disease progression and prompt sight saving treatments as required. This article describes the data contained within the INSIGHT Birmingham, Solihull, and Black Country Diabetic Retinopathy Dataset.

**Design:**

Dataset descriptor for routinely collected eye screening data.

**Participants:**

All diabetic patients aged 12 years and older, attending annual digital retinal photography-based screening within the Birmingham, Solihull, and Black Country Eye Screening Programme.

**Methods:**

The INSIGHT Health Data Research Hub for Eye Health is a National Health Service (NHS)–led ophthalmic bioresource that provides researchers with safe access to anonymized, routinely collected data from contributing NHS hospitals to advance research for patient benefit. This report describes the INSIGHT Birmingham, Solihull, and Black Country DR Screening Dataset, a dataset of anonymized images and linked screening data derived from the United Kingdom’s largest regional DR screening program.

**Main Outcome Measures:**

This dataset consists of routinely collected data from the eye screening program. The data primarily include retinal photographs with the associated DR grading data. Additional data such as corresponding demographic details, information regarding patients’ diabetic status, and visual acuity data are also available. Further details regarding available data points are available in the supplementary information, in addition to the INSIGHT webpage included below.

**Results:**

At the time point of this analysis (December 31, 2019), the dataset comprised 6 202 161 images from 246 180 patients, with a dataset inception date of January 1, 2007. The dataset includes 1 360 547 grading episodes between R0M0 and R3M1.

**Conclusions:**

This dataset descriptor article summarizes the content of the dataset, how it has been curated, and what its potential uses are. Data are available through a structured application process for research studies that support discovery, clinical evidence analyses, and innovation in artificial intelligence technologies for patient benefit. Further information regarding the data repository and contact details can be found at https://www.insight.hdrhub.org/.

**Financial Disclosure(s):**

Proprietary or commercial disclosure may be found after the references.

Diabetes mellitus (DM) affects a significant portion of the population worldwide.^1^^,^^2^ Diabetic retinopathy (DR), one of the most common microvascular complications associated with DM, is a major cause of preventable sight loss globally.^3^ In the United Kingdom (UK), it is projected to affect approximately 4.6 million (9.5%) of the population by 2030.^1^^,^^2^^,^^4^

The Birmingham, Solihull, and Black Country Eye Screening Programme, which commenced in 2007, aims to invite all diabetic patients aged 12 years and above for annual digital retinal photography-based screening. The program fulfills the national Public Health England Diabetic Eye Screening Programme (DESP) requirements and encompasses patients who fall under 9 hospital catchment areas across Birmingham, Dudley, Walsall, and Wolverhampton, within the West Midlands (England, UK), and is hosted by University Hospitals Birmingham (UHB) National Health Service (NHS) Foundation Trust to which the screening data are uploaded. This screening program provides screenings to over 200 000 individuals and includes longitudinal follow-up data up to 15 years; it is thought to be one of the largest urban diabetic screening schemes in Europe.

The INSIGHT Hub aims to maximize the benefits and impact of historical, patient-level NHS hospital admission and electronic health record data by making it research-ready, including curation, pseudonymization, and anonymization. INSIGHT is one of the number of Health Data Research Hubs established by UK Research and Innovation through Health Data Research UK (HDRUK). INSIGHT was formed through a collaboration partnership between the NHS (UHB NHS Foundation Trust and Moorfields Eye Hospital NHS Foundation Trusts), academia (University of Birmingham), industry (Roche, Google), and charity (Action Against Age-Related Macular Degeneration). INSIGHT enables access to anonymized routinely collected patient data from UHB and Moorfields Eye Hospital, focusing on eye health, and the emerging field of "oculomics," in which the eye is used as a "window" into systemic health, including the discovery of novel biomarkers for diseases such as dementia and ischemic heart disease.^5^ Built on the ethically approved INSIGHT research database, the hub has established efficient and robust governance processes that support safe and secure access to anonymized extracts of "evergreen" datasets that are continuously updated in line with the clinical services. One of these datasets is the INSIGHT Birmingham, Solihull, and Black Country DR Dataset,^6^ a research-ready longitudinal record of routinely collected screening data relevant to diabetic eye disease.

In this article, we describe the INSIGHT Birmingham, Solihull, and Black Country DR Dataset by creating a datasheet that utilizes the headings of "motivation, composition, collection process, preprocessing/cleaning/labeling, uses, distribution, and maintenance." This format is adapted from the datasheets for datasets guidance, is outlined by Gebru et al, and has included all sections relevant to the INSIGHT Birmingham, Solihull, and Black Country DR Dataset.^7^^,^^8^

## Datasheet

### Motivation for Dataset Creation

Diabetic retinopathy is a major cause of visual deficit worldwide and a leading cause of blindness in the working age population.^3^ Diabetic retinopathy is the most common microvascular complication associated with DM, affecting approximately 40% of patients with diabetes.^9^ The pathogenesis of DR involves microangiopathy and capillary occlusion leading to retinal ischemia and an increase in vascular endothelial growth factor levels. As a result, macular edema and retinal neovascularization are responsible for sight loss.^3^^,^^10^^,^^11^ Although laser and surgical interventions, such as panretinal and focal retinal photocoagulation, are available for advanced neovascular DR, the prevention of disease and its progression is vital.^12^ Blood pressure control and, more importantly, tight glycemic control are important sight-preserving primary prevention measures.^13^

In conjunction with preventative measures, early detection and prompt treatment of DR are important to minimize visual loss. The St. Vincent Declaration in 1989 stated that a primary objective for Europe should be a reduction in diabetes-related blindness by at least one third.^14^ In response to the high burden of diabetes-associated ocular morbidity, the NHS DESP for England was initiated in 2003 with the primary objective being to reduce sight loss among the diabetic population through early detection.

Advances in computing power and the field of machine learning have introduced new avenues of research in health care, particularly in diagnostic systems. The use of artificial intelligence for the detection of DR has been illustrated in the literature, with studies showing promising evidence for a potential transition automated screening in the future.^15^, ^16^, ^17^, ^18^ A key barrier to artificial intelligence training and validation is a shortage of datasets containing sufficient volumes of data with reference standards and accurate labeling In addition to artificial intelligence development, datasets can be utilized for the development of other novel diagnostic and interventional technologies. The main advantages of DR data include the following:

• Large volumes of routinely collected longitudinal data obtained from an ethnically diverse population representing an entire region of England
• Accurate ground truth data from a nationally endorsed screening program with robust processes for participant inclusion and quality management of data

### Dataset Composition

The Birmingham, Solihull, and Black Country DESP is set within the West Midlands. The region includes a diverse ethnic and socioeconomic mix with a higher than UK average of minority ethnic groups. There are particularly high rates of diabetes, physical inactivity, obesity, and smoking in this region.

The INSIGHT Birmingham, Solihull, and Black Country DR Dataset is routinely collected data from the eye screening program and comprises data relating to multiple episodes for each patient. Each episode (patient visit) includes the retinal photograph along with corresponding demographic and DR grading data. The dataset contains all instances collected via the eye screening program from its inception to present day. Images are graded to output a retinopathy and maculopathy score as shown in Table 1. A grade is assigned for retinopathy (R) and maculopathy (M); for example, "R0M0" would signify that the patient has no DR at the time the images were graded. Patients receive a grade based on the eye with the most advanced DR and are directed into the appropriate pathway.^19^

**Table 2:** Demographic Data for Patients Included Within This Dataset

Demographic	Value	Frequency	Proportion
Sex	Male	129 927	52.78%
	Female	109 321	44.41%
	Unknown	6932	2.82%
Ethnicity	British	125 553	51.00%
	Unknown	31 980	12.99%
	Pakistani	25 461	10.34%
	Indian	23 676	9.62%
	Caribbean	10 474	4.25%
	Any other White background	6090	2.47%
	Bangladeshi	5437	2.21%
	African	4039	1.64%
	Any other Asian background	3714	1.51%
	Irish	2481	1.01%
	Any other Ethnic group	2307	0.94%
	White and Black Caribbean	1438	0.58%
	Any other Black background	1380	0.56%
	Chinese	848	0.34%
	Any other mixed background	469	0.19%
	White and Asian	436	0.18%
	White and Black African	397	0.16%
Age (yrs)	< 20	3442	1.40%
	21–30	5441	2.21%
	31–40	16 977	6.90%
	41–50	39 161	15.91%
	51–60	56 017	22.75%
	61–70	57 960	23.54%
	71–80	47 437	19.27%
	81–90	18 105	7.35%
	91–100	1628	0.66%
	101–110	12	0.00%

Demographic details included here are those recorded at patients’ first presentation. For example, if a patient was aged 30 years at their first attendance to the screening service, then they would be in the 21–30 years age bracket in the table above.

The dataset has been kept updated with new patient encounters via the screening program. Table 2 illustrates demographic data representing the patients who have been seen in the screening program (based on all cases registered between January 1, 2007, and December 31, 2019). The age of participants displayed in Table 2 is the age at first encounter (first visit screening appointment with the service). Over the past few years, a drive has been made to reduce missing ethnicity data by obtaining ethnicity from primary care systems for active patients. In currently available data, 87% of individuals have ethnicity recorded (with 13% being unknown).

**Table 1:** Table Showing Routine Digital Screening Grades by Year of Screening Including Retinopathy (R) and Maculopathy (M) Grades

Retinal Degeneration Slow Grades by Year (Original + Inferred)																		
Year	R0 M0 (No DR)	DR (Any Grade)∗	R1 M0	R1 M1	R2 M0	R2 M1	R3 M0	R3 M1	R3A M0	R3A M1	R3S M0	R3S M1	Incomplete Grading Data	Inadequate Image	Ungradable Image	No. Photos	No. Grade Information	Total
2007	30 484	14 036	10 034	2919	272	389	197	225	0	0	0	0	1287	476	189	91	12	46 575
2008	48 867	25 886	18 504	5451	408	590	362	571	0	0	0	0	568	436	483	0	26	76 266
2009	57 388	30 231	22 833	5258	474	670	404	592	0	0	0	0	359	580	596	1	20	89 175
2010	59 981	32 831	23 670	5829	679	1010	563	1080	0	0	0	0	135	1154	664	47	5	94 817
2011	62 142	33 944	25 903	5424	726	932	354	605	0	0	0	0	0	4173	91	309	0	100 659
2012	69 278	32 492	25 362	4973	619	698	350	490	0	0	0	0	10	3549	106	332	0	105 767
2013	78 567	32 031	25 491	4570	551	642	359	418	0	0	0	0	3	3434	128	377	0	114 540
2014	82 993	32 415	24 958	5459	468	674	306	359	32	59	64	36	0	3782	175	320	0	119 685
2015	87 341	32 156	22 813	7076	412	712	0	0	182	401	330	230	8	4045	530	0	0	124 080
2016	91 385	33 746	23 351	7804	466	716	0	0	177	384	540	308	10	3583	590	0	0	129 314
2017	92 367	33 463	22 804	8027	430	784	0	0	152	395	595	276	8	3501	594	0	0	129 933
2018	97 764	34 548	23 420	8027	547	925	0	0	184	453	657	335	14	3253	634	0	0	136 213
2019	98 326	35 885	24 955	8107	521	882	0	0	193	366	584	277	8	3054	648	0	0	137 921
Total	956 883	403 664	294 098	78 924	6573	9624	2895	4340	920	2058	2770	1462	2410	35 020	5428	1477	63	1404 945

Incomplete grading data includes grades with a retinopathy label but no maculopathy label and vice versa. Inadequate images are those in which the image itself may be of reduced quality because of things such as media opacities (for example, cataracts), but the grading can still occur. Ungradable images are those in which grading was considered inappropriate because the images were so poor (for example, due to patient factors such as movement, etc.). In 2014, the new grades of R3S (stable) and R3A (active) were introduced. DR = diabetic retinopathy.

∗ Values in the DR (any grade) column marked with an asterisk are not included in the totals column on the right-hand side of the table. This column contains totals of all grades from R1M0 to R3SM1.

**Figure 1:**
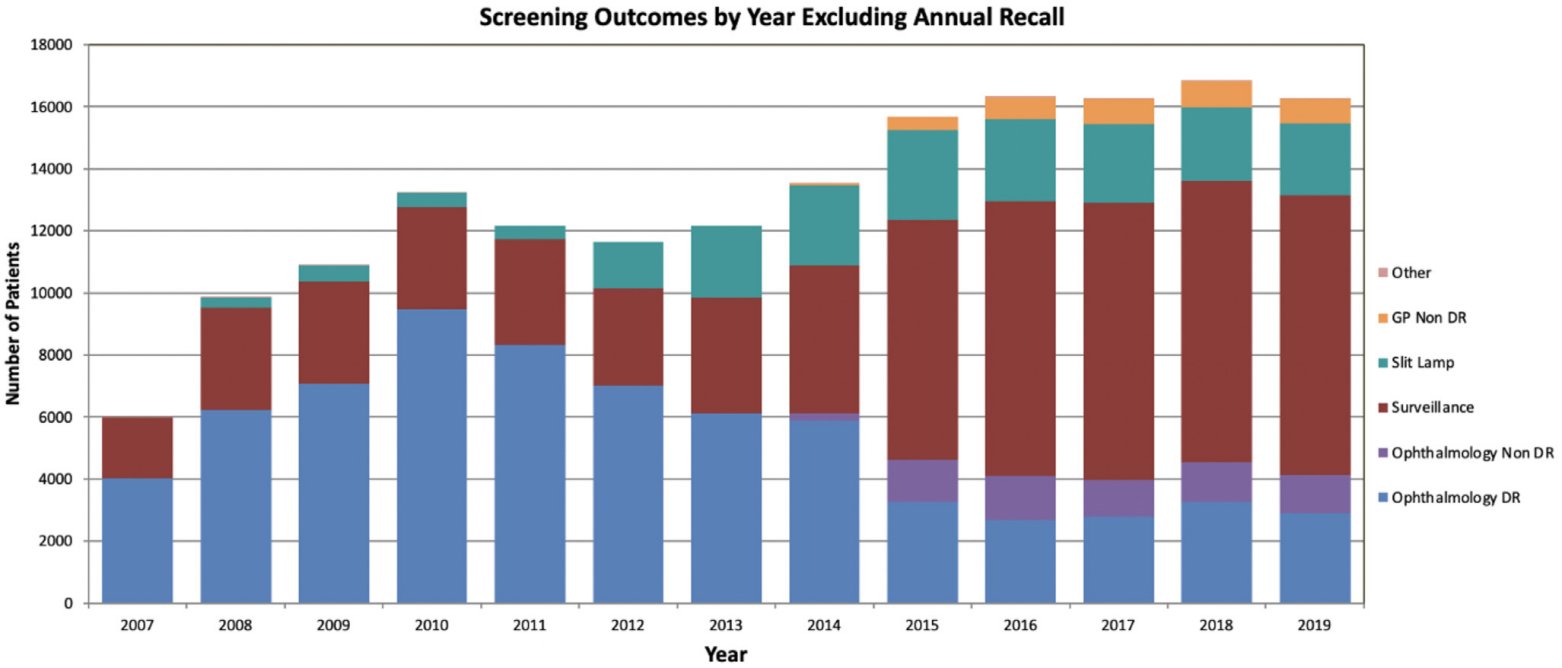
Bar graph showing the number of patients seen per year from 2007 to 2019. DR = diabetic retinopathy; GP = general practice.

**Figure 2:**
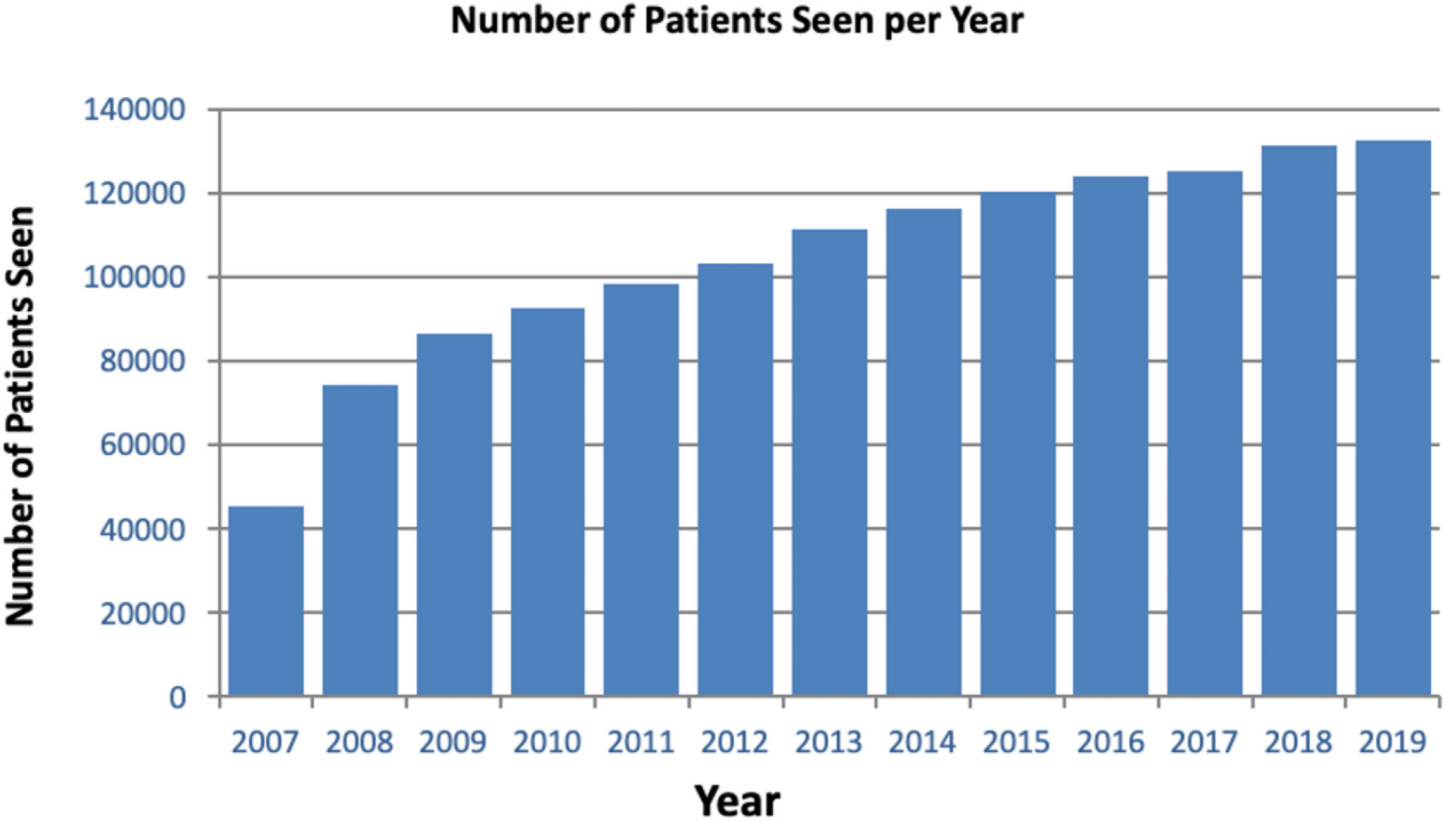
Bar graph showing the total number of images obtained through the eye screening program per year from 2007 to 2019.

**Figure 3:**
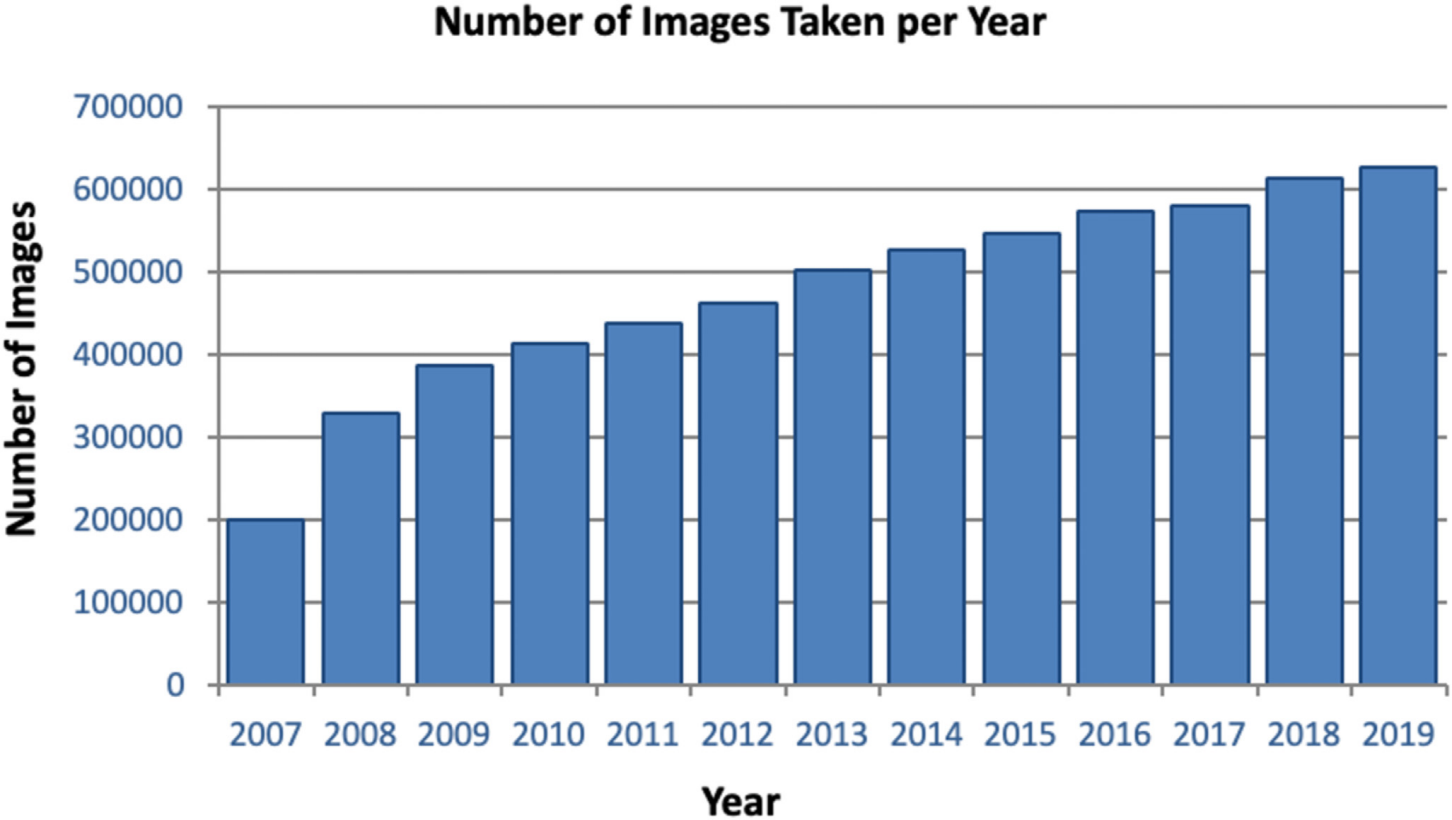
Bar graph showing screening outcome distribution per year from 2007 to 2019. This graph does not include patients whose outcome was annual recall (routine digital screening). Screening outcomes are described in further detail in the section Screening Outcomes.

**Figure 4:**
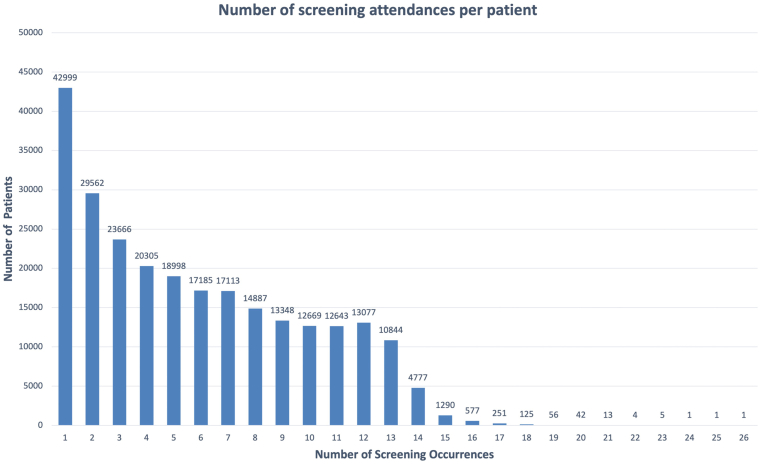
Bar chart showing the availability of longitudinal data. The number of screening occurrences is plotted against the number of patients. For example, there are 12 669 patients for whom data relating to 10 screening visits are available.

The analysis described here reflects a time-locked data extract as of December 31, 2019, comprising data collected between January 1, 2007, and December 31, 2019, hereafter referred to as INSIGHT Birmingham, Black Country, and Solihull DR Dataset Extract 2007–2019. From 2007 to 2019, the dataset includes 6 202 161 images from 246 180 individuals (Figure 1, Figure 2, Figure 3). Figure 4 shows a breakdown of patients and images by year. Key data included in the dataset are as follows:

• Total number of patients screened and graded over a 13-year period
• Demographic information (including age, sex, and patient reported ethnicity)
• Diabetes status
• Diabetes type
• Length of time since diagnosis of diabetes
• Visual acuity
• The national screening diabetic screening grade category (7 categories from R0M0 to R3M1)
• Diabetic eye clinical features
• Reason for sight impairment and severe sight impairment
• Screening outcome (digital surveillance and time; referral to hospital eye service [HES])

A full description of data points that are available are listed in Supplementary File 1. An online data dictionary is available at https://web.www.healthdatagateway.org/dataset/36886b21-12ff-45e7-82bc-fb5308c12450. There are a number of data points that are essential to be reported (such as demographics, visual acuity, grading, and triage outcome). Some fields are mandatory to fill, such as grade, and other fields have optional manual input, such as diabetes type. The screening service data system has evolved over the years of operation, with the addition and removal of fields; these fields are, therefore, limited in their completeness. Data points are linked longitudinally between patient encounters to enable the tracking of disease progression.

Grading labels are assigned through the eye screening program, and this process is described in detail in the next section. The INSIGHT Birmingham, Black Country, and Solihull DR Dataset Extract 2007–2019 comprises 1 360 547 grading episodes between R0M0 and R3M1, with 44 335 grading episodes having incomplete grading data, inadequate images, ungradable images, or no photos. Table 1 shows the composition of the dataset by DR grade and year.

The dataset is composed of routinely collected data that represent routine clinical processes within the eye screening program. Given that the screening service aims to offer appointments to all patients in the area with DR over the age of 12 years, there is a high level of inclusion; however, it is recognized at a national level that there is reduced engagement with the screening service by certain groups and that, despite being a free health service, some groups may experience barriers to access, such as transport, caring or work duties, or the need for a carer to accompany them.

Although this dataset is self-contained, INSIGHT provides the capability to provide linked health data on those individuals within this dataset who also attend UHB, whether for hospital eye care or for other systemic health issues. This more holistic record can support the discovery of new associations between retinal status and systemic health in people with diabetes. Researchers can, therefore, apply to access datasets that represent patient care within the eye screening program alone; or patient care combining the screening service and their linked UHB hospital eye care^20^; or patient care comprising the screening service data and acute UHB diabetic hospital admissions^20^ and/or a range of other systemic comorbidities and outcomes, such as UHB cardiac outcomes.^20^ As an example, when the DR screening data were linked to the UHB hospital cardiac outcomes, the DR screening-cardiac outcome dataset comprised 1 760 093 eye images from 61 252 individuals with 272 863 *International Classification of Diseases, 10th Revision* cardiac codes and 10 693 Systematized Nomenclature of Medicine cardiac episode codes.^21^^,^^22^

### Collection Process

The collection process is described as it applies to the Birmingham, Solihull, and Black Country Eye Screening Programme 2007–2019 dataset and as it continues to be undertaken at the time of writing (September 1, 2022); it cannot be assumed to continue unchanged beyond this date because this will reflect both local and national guidance. The collection process is summarized in 4 sections. The first focuses on the historical development of the screening service and screening pathways, the second outlines the types of screening categories that differ from routine digital screening annually, the third describes image capture, and the fourth describes the grading (image labeling) pathway.

#### Birmingham, Solihull, and Black Country DESP

The DESP started in 2007, centered on Birmingham Heartlands Hospital, with a total of 6 hospitals participating in 2007, an additional hospital joining in 2008, and 2 Black Country hospitals joining in 2014 (one of which onboarded retrospective data from 2007). The screening program operates on an optometry-based model with 9 contributing HESs. Ninety sites were originally involved; this has increased with time to 110 screening sites.

All patients with diabetes aged above 12 years are offered yearly diabetic eye screening nationally. Over 130 000 patients were screened in 2019 with almost 80% of eligible patients responding to an invitation for screening. If the person has no retinopathy, they are returned to annual screening; however, if they have preproliferative or active proliferative retinopathy, patients are referred on for further assessment and treatment in HESs.

#### Screening Outcomes

There are 5 screening outcomes in this dataset aside from those in routine digital screening annually:

• Surveillance pathway for DR
• Surveillance with slit-lamp biomicroscopy
• General practice (GP) non-DR (those referred back to primary care)
• Ophthalmology DR
• Ophthalmology non-DR

*Surveillance* screening is additional in the year screening for any patient who has changes in the retina, changes that may develop or disappear in due course but if left for 12 months could potentially lead to a change in the management plan such, as the need for treatment. A patient would have their normal annual screen and be referred through to surveillance rather than HES for extra attention within the screening year. The surveillance category was developed in Birmingham by Professor Paul Dodson in the early 2000s and recommended by the National Screening Service in 2010. In effect, it allows closer monitoring of patients in the hospital setting without the need to refer through to the ophthalmology clinics in the HES, thereby reducing the HES workload and being a more cost effective measure. Surveillance clinics are staffed by trained screener/graders and the images graded by senior graders, with options including return to annual screening, screen again within a determined number of months, and refer to HES.

*Surveillance withslit-lampbiomicroscopy* usually follows an annual screening instance in which images are ungradable (U); ungradable images are ones in which it is inappropriate to grade because the images are so poor (for example, due to patient factors such as movement). A transition period between 2014 and 2015 can be observed where there was an increase in the number of ungradable images (Table 1). This was due to a change in the process that meant that it was impossible to send a patient from annual screening without submitting images. If the image was ungradable, often an anterior shot would be taken, providing evidence that the equipment was not dysfunctional. This process enabled reimbursement of the screening visit. Surveillance with slit lamp takes place within an HES clinic by senior screeners trained to perform dilated fundoscopy using slit-lamp biomicroscopy; it is purely a clinical assessment by the screener, with no images being recorded.

*General practicenon-DR* patients are those who are seen in the DESP but do not have DR and so are referred back to primary care for future care.

*Ophthalmology DR* patients are those who are referred to HESs for ophthalmology appointments. Those in the category for *ophthalmology* *non*-*DR* are those who are referred to the HESs for other eye conditions, such as suspected glaucoma, cataract, and so on.

Within the active patients in the screening program in 2019, patient outcomes included 121 667 for annual recall. A total of 9033 patients were sent to the surveillance pathway for DR; 2302 patients were sent to slit-lamp biomicroscopy surveillance; 2920 patients were referred to Ophthalmology for DR; 1209 were referred to Ophthalmology for non-DR ophthalmic conditions; and 788 patients were referred back to their GPs due to not having DR (Fig 1). A proportion of patients have the screening outcome "other." The "other" category includes patients with screening outcomes that do not fit in the 5 outcome categories. Outcome data for these patients (other) are unavailable.

Pregnancy DR screening data are also available within this dataset and are available to be requested from INSIGHT. The national requirement is to offer screening after the first antenatal clinical appointment.^23^ If DR is detected, then an additional retinal assessment is offered between 16 and 20 weeks. Another retinal assessment is recommended at 28 weeks with referral onward to the HES as necessary.

#### Image Capture

Images are captured using retinal photography and transferred to the centralized grading center at Birmingham Heartlands Hospital, which is part of UHB NHS Trust. The majority of the retinal cameras in use are Topcon cameras (TRC-NW6), with other cameras including Canon CR-DGi, Nidek AFC-210, and Kowa Alpha 8. Once images have been captured, they are ingested into OptoMize (Digital Healthcare Limited, UK) and stored in a Structured Query Language database hosted by a client server within UHB.

#### Grading Pathway

The grading pathway is supported by OptoMize and is conducted using a "feature-based" assessment. Before 2018, images were screened by graders who made decisions regarding DR status. Following this, graders are now responsible for identifying and entering retinal features into the OptoMize software, which uses a rule-based decision system to generate the grading outcome. Retinal photographs are queued by the patient identifier in the software and screened by graders chronologically. If patients are deemed to have background DR (R1) or worse, then the images are forwarded to a second grader who will identify features independently of the first grader. The OptoMize software then decides whether patients will need to be referred to a more experienced arbitration grader, who will see the first 2 grading outcomes and make an informed judgment on the final grade outcome. Additionally, quality control measures ensure 10% of patients with no DR, who will be referred to a secondary grader. This grading process, involving manual validation and quality control, ensures that data capture is accurate.

This grading pathway, as part of the screening program, generates data labels for the image set within the INSIGHT Birmingham, Solihull, and Black Country DR Dataset. The image set would include at least 1 disc-centered and 1 macula-centered image of adequate quality per eye. It is important to note that the grade is assigned per eye rather than by the individual image. This means that grades cannot currently be provided on a per image basis. The final referral outcome is based on the worst grade of the 2 eyes.

### Ethics

The INSIGHT Birmingham, Solihull, and Black Country DR Dataset is created through the INSIGHT research database, which was approved by the West of Scotland Research Ethics Committee 4 in 2020 (ref: 20/WS/0087), and received all institutional governance approvals in the same year. A commitment to use routinely collected data in anonymized form "to support research and improve care for others" is enshrined within the NHS Constitution.^24^ INSIGHT is one of a number of UK initiatives that support this within a strict governance framework and with patient and public involvement to provide independent oversight of the data access processes.^25^ A fuller description is provided elsewhere,^26^ but in brief, (1) INSIGHT respects the request of any patients who do not wish their data used in this way and has robust processes working with NHS Digital to ensure that no data are included within the research database from individuals who have exercised their right to opt out using the NHS Digital National Data Opt-Out Service.^27^ The associated NHS trusts actively promote awareness of their proposed use of the data among their patient communities, and of the option to opt out (including instructions on how to do so). (2) Independent review of data use applications to INSIGHT is conducted by the INSIGHT Data Trust Advisory Board, which comprises independent membership of patients, public, and sector experts; the Data Trust Advisory Board advisory recommendation to the data controller (in this case, UHB) is critical to any decision to provide data access. (3) Patients and the public are also involved in the processes informing the development of the INSIGHT Hub and through 2 lay advisors who are members of the INSIGHT Leadership Group.

### Preprocessing/Cleaning/Labeling

Images are processed using INSIGHT cloud-based technology in pairs, consisting of a full-sized image along with the corresponding thumbnail. Full images contain Exchangeable Image File (EXIF) data and are high resolution, whereas the thumbnails are smaller sized files with no EXIF data. Full images are pushed through EXIF processing and through conversion to Digital Imaging and Communications in Medicine (DICOM) (or other required image format) using Moorfields Librarian, a custom-built software tool created by Softwire Technology Limited (London, UK) for Moorfields Eye Hospital NHS Foundation Trust. This custom-built tool is not open source, and the code is owned by Softwire. Because of the volume of data being transferred into the UHB environment, quality control was integrated into the preprocessing pipeline. The file type and content of the image are validated using automated tooling developed for preprocessing, so each image is individually validated. Thumbnails are pushed through 2 deep learning classifiers developed within UHB: (1) the "sorting hat model" and (2) the "laterality and fixation model."

#### Thumbnail Processing

##### Identifying Retinal Images

The sorting hat model is a deep learning classifier that was developed to distinguish anterior eye images and posterior retinal fundus images (area under the curve [AUC] > 99.9%) to provide a pure retinal image dataset for DR studies. The model is written in Python 3.8 and built on the TensorFlow 2.0 framework.

##### Identifying Laterality and Fixation

Laterality and fixation are useful to researchers but are not mandatory fields for the DR screening service and are, therefore, not always recorded by the human graders. Laterality is recorded in approximately 65% of images and fixation in approximately 7% of images. INSIGHT constructed the laterality and fixation model, which runs on the retinal images output by the sorting hat model. This model is used to label the image laterality (left eye vs. right eye; AUC, 99.08%) and whether the retinal image was disc-fixated or macular-fixated (AUC, 99.27%). This model is written in Python 3.8 and built on the TensorFlow 2.0 framework.

#### Full Image Processing

##### EXIF Processes

Over 95% of full-sized images are obtained in Joint Photographic Experts Group (JPEG) format. They are first run through the EXIF processes. All EXIF processes use EXIFTOOL, which is publicly available (https://exiftool.org/). The “EXIF grabber” reads and stores all of the EXIF data. The EXIF tags for images include camera information, camera setting information, and image settings. The “EXIF stripper” is then used to strip all EXIF data from the images, to remove any nonessential unique data, and so to reduce any risk of reidentification.

##### Image Conversion (Including DICOMization)

The next step in full image processing involves use of the Librarian application, which converts images from JPEG into DICOM format. Although most images ingested into the INSIGHT hub from the DESP are in JPEG format, some images are formatted to Portable Network Graphic. The Python Imaging Library package in Python is used to convert Portable Network Graphics to JPEGs using quality at 75 (Python Imaging Library default) before the images are converted to DICOM using Librarian. As the images are validated on ingress, the main quality control on egress is to ensure that DICOM images remain uncorrupted. Manual validation of the Librarian software was completed during the testing and development phase in addition to the initial production phase. Quality assurance is also performed on images during extraction for fulfillment of a data request.

The INSIGHT Birmingham, Solihull, and Black Country DR Dataset is available in both JPEG and DICOM formats. Image pixel data are not altered during the processes described above.

### Uses

The INSIGHT Birmingham, Solihull, and Black Country DR Dataset has been prepared to support research for patient benefit including from discovery to validation using both tabular and image data. Specific examples include discovery of novel associations with DR, exploration of health disparities, analysis of trends over time, development of artificial intelligence as a medical device tool for DR classification, and validation of these tools across populations, including as part of regulatory applications. In addition, when linked to systemic data, applications include the identification of retinal biomarkers of systemic disease in the context of diabetes including cardiovascular or cerebrovascular event, renal failure, peripheral vascular disease, peripheral neuropathy, foot ulcers, anemia, dementia, or another systemic health output that would be routinely collected within routinely collected hospital data. Publications using INSIGHT data will be cited on the website to illustrate examples of data use. The dataset description may be viewed (and access applied for) on the INSIGHT website and via the HDRUK Innovation Gateway.^28^

### Distribution

Data use applications for the INSIGHT Birmingham, Solihull, and Black Country DR Dataset can be made via the HDRUK Innovation Gateway or by contacting the hub directly (www.insight.hdrhub.org). The data use application form includes a description of the researcher, a plain English summary of the project, the expected public benefit and a detailed description of how the data will be used, and the data use environment. Applications are welcomed from all bona fide researchers representing recognized research organizations with a clear commitment to patient benefit. All research data applicants should be able to demonstrate information security and health data research best practice.^29^, ^30^, ^31^, ^32^, ^33^, ^34^, ^35^

Data use applications undergo sequential stages of evaluation: first, internal INSIGHT checks, including due diligence (applicant/institution) and evaluation of whether the dataset is suitable for the proposed project; second, review by the Data Trust Advisory Board, providing independent advisory recommendation regarding the use of data and anticipated patient and public benefit; third, evaluation by the data controller (in this case, UHB) who has the legal responsibility and makes the final decision.^26^ If the application is supported, the data controller and the applicant proceed to contractual discussions, including agreeing on access arrangements and financial terms that secure a sustainability and fair value return to the NHS. The contractual discussions normally take the form of establishing a data license agreement.

The data controller may determine how access to the data is made available, which is, in this case, through the UHB Trusted Research Environment, or an equivalently secure data environment that has been approved by the data controller. The Trusted Research Environment is a provisioned cloud environment. Data cannot be downloaded for local use, and application programming interfaces are not available for data access. The data are provided in DICOM format for retinal images and in comma-separated value for tabular data. JavaScript Object Notation and YAML (yet another markup language) formats are not available.

In addition to complying with all UK General Data Protection Regulation and Data Protection law and best practice, the INSIGHT Data Use Application process aligns to the “5 Safes” framework (Welpton TDFR, unpublished data, 2016):^36^

1. Safe data: data are treated to protect any confidentiality concerns.
2. Safe projects: research projects are approved by data owners for the public good.
3. Safe people: researchers are trained and authorized to use data safely.
4. Safe settings: a secure research environment prevents unauthorized use.
5. Safe outputs: screened and approved outputs that are nondisclosive.

### Strengths and Limitations

Strengths of this dataset (and indeed other datasets available through INSIGHT) are their scale, their richness, and their diversity. There are a number of publicly accessible DR datasets available globally.^37^, ^38^, ^39^ These datasets vary in size, with Nagpal et al^38^ identifying datasets containing 16 to 9963 fundus images. Furthermore, the majority of these datasets are not routinely updated with contemporary clinical data. The INSIGHT dataset contains over 6 million images with up to 15 years of follow-up data. Lastly, linked systemic data can be requested through INSIGHT, allowing for the investigation of new associations between eye and systemic health.

The main limitations of the INSIGHT dataset are those common to real-world datasets, including the completeness of data and the level of quality assurance when compared with datasets from well-conducted clinical trials; however, this is mitigated by the dataset being derived from a quality-assured diabetic screening service with a high level of verification of image labels. An additional limitation is that there is no pixel-specific annotation of images. In comparison, Porwal et al^39^ describe pixel-level annotation data, allowing those without specialist retinal expertise to train algorithms.

The dataset extraction described here runs up to December 31, 2019. The coronavirus disease 2019 pandemic had a significant impact on health services globally, including the UK.^40^ The DR screening service was modified in response to the pandemic, and patients that were in annual recall in 2019 (graded R0M0) were not screened in 2020, with a screen scheduled for 2021. There are plans ahead for a potential 2-year recall pathway for routine screening. In light of the impact of the pandemic and subsequent changes to the pathway, we limited the data extract reported here to the period from 2007 to 2019 exclusively. Data after this period are also available for research purposes through INSIGHT but are not described in this paper.

## Summary

This article describes the INSIGHT Birmingham, Solihull, and Black Country DR Dataset, including a detailed description of the 2007 to 2019 data extract.^41^ This dataset is a large-scale, updating, anonymized data resource generated securely from routinely collected NHS data (specifically the Birmingham, Solihull, and Black Country DESP). The dataset comprises over 6 million retinal photographs and relevant longitudinal clinical data, with capability to include other relevant systemic health data where appropriate. Access to the dataset is provided by the data controller (UHB) through the INSIGHT Health Data Research Hub. This datasheet provides a summary of the dataset to encourage transparency in dataset creation and the development of novel technologies, in addition to enhancing communication between dataset creators and users. Further information and contact details can be found at https://www.insight.hdrhub.org/.
